# Josiah Bacon’s Death

**Published:** 1879-05

**Authors:** 


### JOSIAH BACON'S DEATH.

On Sunday, April 13th, at about twelve o'clock, m., Josiah Bacon was found dead on the floor in his room at the Baldwin Hotel, San Francisco.

There was no blood on the floor or the clothes of the dead man, and when found the body was cold and rigid, having been dead probably three hours.

The agent of the deed, and the cause was, at first, shrouded in mystery, but on the succeeding Wednesday the whole matter was revealed by the appearance of Dr. S. P. Chalfant, of that city, who walked into the central police station and surrendered himself, and acknowledged that he had killed Mr. Bacon, but claimed that it was accidental. Dr. Chalfant had been subjected to rigorous prosecution several different times, for alleged infringement of the Goodyear Dental Vulcanite Company's patent, in the use of rubber for artificial teeth.

Upon this subject Dr. Chalfant had called to see Mr. Bacon. The interview became very heated, when Dr. Chalfant drew his pistol, not to shoot, but to intimidate Mr. Bacon, the weapon accidentally was discharged, and Mr. Bacon was killed.

It may never be known whether this act was accidental or intentional, but in either case there are certain facts that can not be overlooked.

Mr. Bacon had prosecuted and broken up Dr. Chalfant's business and virtually drove him from his field of practice twice before. Mr. Bacon said to Deputy Marshall McEwan, on the Friday before his death, when asked, "Do you ever push dentists to the wall." "Certainly we do when they don't pay up. There is Chalfant, for instance, I found him in Wilmington, Del., using our patent without having paid the royalty. I commenced action against him, got out an injunction, obtained judgment, upon which an execution was issued, and broke him up. He then left town. When I went to St. Louis I found him engaged in the same business, and I broke him up again. I find him here doing the same thing, and he will have to pay or be broken up again."

These words indicate about Mr. Bacon's course with dentists generally. They had to submit to his demands or suffer prosecution, and he was by no means a mild prosecutor.

The manner in which Dr. Chalfant had been followed up had doubtless brought him almost, if not quite, to desperation. And, if reports be correct, he was far from being alone in this respect. The affair was one much to be regretted, both n the occasion and the occurrence.

